# Correlation between Chemical Composition of *Curcuma domestica* and *Curcuma xanthorrhiza* and Their Antioxidant Effect on Human Low-Density Lipoprotein Oxidation

**DOI:** 10.1155/2012/438356

**Published:** 2012-11-26

**Authors:** Ibrahim Jantan, Fadlina Chany Saputri, Muhammad Naeem Qaisar, Fhataheya Buang

**Affiliations:** ^1^Drug and Herbal Research Center, Faculty of Pharmacy, Universiti Kebangsaan Malaysia, Jalan Raja Muda Abdul Aziz, 50300 Kuala Lumpur, Malaysia; ^2^Faculty of Pharmacy, Universitas Indonesia, Kampus UI, Depok 16424, Indonesia; ^3^Faculty of Pharmacy, University of Sargodha, University Road, Sargodha, Lahore 40100, Pakistan

## Abstract

The antioxidant activity of the curcuminoids of *Curcuma domestica* L. and *C. xanthorrhiza* Roxb. and eight compounds which are prevalent constituents of their rhizome oils were investigated in an effort to correlate human low-density lipoprotein (LDL) antioxidant activity with the effect of the herbs and their components. The antioxidant activity was examined using thiobarbituric acid reactive substances (TBARSs) assay with human LDL as the oxidation substrate. The methanol extracts and rhizome oils of *C. xanthorrhiza* and *C. domestica* showed strong inhibitory activity on copper-mediated oxidation of LDL. Curcumin, demethoxycurcumin, and bisdemethoxycurcumin, isolated from the methanol extracts of both plants, exhibited stronger activity than probucol (IC_50_ value 0.57 **μ**mol/L) as reference, with IC_50_ values ranging from 0.15 to 0.33 **μ**mol/L. Xanthorrhizol, the most abundant component (31.9%) of the oil of *C. xanthorrhiza*, showed relatively strong activity with an IC_50_ value of 1.93 **μ**mol/L. The major components of *C. domestica*, ar-turmerone (45.8%) and zerumbone (3.5%), exhibited IC_50_ values of 10.18 and 24.90 **μ**mol/L, respectively. The high levels of curcuminoids in the methanol extracts and xanthorrhizol, ar-turmerone and zerumbone in the oils, and in combination with the minor components were responsible for the high LDL antioxidant activity of the herbs.

## 1. Introduction

*Curcuma* (Zingiberaceae) is a large genus of rhizomatous herbs distributed in tropical and subtropical regions especially in India, Thailand, the Malay Archipelago, Indochina, and Northern Australia. Many species have been cultivated, and their powdered rhizomes have been widely used as flavours in native dishes and ingredients in many traditional medicines to treat various ailments [1]. The more popular and economically more important species, *C. domestica* L. and *C. xanthorrhiza* Roxb., are more widely used as condiments than for their medicinal purposes. Many phytochemical studies on the extracts and essential oils of several *Curcuma *species, especially *C. longa*, have identified curcuminoids and sesquiterpenoids as the major components [2–6], and these compounds have been identified as the major groups of antioxidants in the plants [7, 8].

 The antioxidant activity of *Curcuma* species, especially *C. longa,* has been measured by various chemical methods such as DPPH radical scavenging activity assay, superoxide anion radical scavenging activity assay, ferric reducing/antioxidant power (FRAP) assay, and metal chelating activity assay [8, 9]. Inhibitory activity of curcumin from *C. longa* and its analogues against free radical initiated peroxidation of human low-density lipoprotein (LDL) [10] and lipid peroxidation and protein oxidation in rat liver mitochondria have been reported [11]. The need to use different methods of antioxidant capacity measurement is due to the various mechanisms of antioxidant action. Determination of the antioxidant activity of plant extracts and compounds often gave different results as the methods used are based on different reaction mechanisms [12]. Although the chemical constituents of *Curcuma* species have been reported and their antioxidant activity has been demonstrated, there has been little effort to correlate the chemical constituents and their antioxidant activity, and the actual substances contributing to the antioxidant activity have not been identified. Direct evidence of therapeutic benefits of the plants and their compounds in cardiovascular disorders remains sparse, and data on LDL oxidation have been few. 

**Figure 1:**
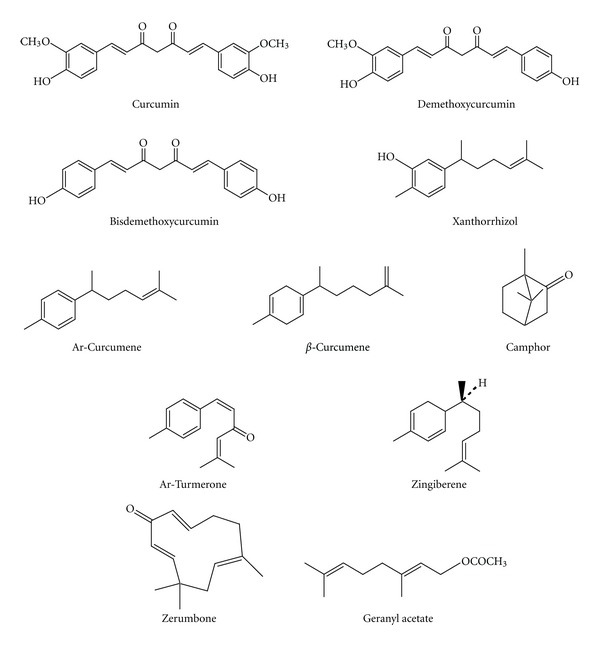
Structures of major compounds from *Curcuma domestica* and *Curcuma xanthorrhiza. *

 In a search for sources of natural cardiovascular protective agents for pharmaceutical, food, and nutraceutical applications, we investigated the antioxidant effect of the methanol extracts and essential oils of *C. domestica* and *C. xanthorrhiza*. The antioxidant activity was determined against copper-mediated isolated human LDL oxidation. Three major curcuminoids, that is, curcumin, demethoxycurcumin, and bisdemethoxycurcumin, were isolated from the methanol extract of the rhizomes of both herbs. The chemical composition of the oils was analysed by GC and GC-MS. The antioxidant activity of the three curcuminoids and eight known constituents (xanthorrhizol, ar-turmerone, camphor, geranyl acetate, zerumbone, *β*-curcumene, zingiberene, and ar-curcumene) of the essential oils of the *Curcuma* species were also investigated in an effort to correlate the effectiveness of the herbs with those of their components. The structures of the major compounds are shown in Figure 1.

## 2. Materials and Methods 

### 2.1. Chemicals and Reagents

The chemicals used in this study were of analytical grade that include methanol, dimethyl sulfoxide (DMSO) and sodium citrate (Merck, Darmstadt, Germany). Camphor, geranyl acetate, zerumbone, *β*-curcumene, zingiberene, ar-curcumene OptiPrep, Sudan Black B, phosphate buffer saline (PBS) tablet, probucol, and Protein Kit were purchased from Sigma Chemical Co. (St. Louis, MO, USA). Subcell electrophoresis and agarose gel were obtained from Bio-Rad, USA. TBARS kit was purchased from ZeptoMetrix Corporation, New York. Melting points were determined using Electrothermal Melting Point Apparatus Model 9100. Ultraviolet (UV) spectra were obtained on Shimadzu UV-1601 spectrophotometer (Shimadzu Corporation, Japan). Molecular weights of the compounds were recorded by ESIMS using ESI-TOFF MS (Bruker MicroToF-Q 86, Switzerland). IR spectra were recorded on a Perkin-Elmer GX spectrophotometer (Massachusetts, USA). The ^1^H and ^13^C spectra were carried out on a JOEL NMR 400 MHz (JOEL Ltd., Japan) with TMS as internal standard. 

### 2.2. Plant Materials and Sample Preparation 

The rhizomes of *Curcuma domestica* and *C. xanthorrhiza* were collected from Kuala Selangor in peninsular Malaysia in the months of March and July 2008. The voucher specimens of *C. domestica* (B-29789) and *C. xanthorrhiza* (B-29783) were identified by Dr. Abdul Latiff Mohamad of Universiti Kebangsaan Malaysia (UKM) and deposited at the Herbarium of UKM, Bangi, Malaysia. The fresh rhizomes of *C. domestica* and *C. xanthorrhiza* were allowed to dry under shade. Five hundred g of dried material of each plant sample were ground and macerated in methanol at the ratio of 1 : 10 (w/v). The extracts were filtered through Whatman No. 1 filter paper, and the entire extraction process was repeated twice on the residue. The filtrates were combined and the methanol was removed under reduced pressure to obtain extracts of *C. domestica* and *C. xanthorrhiza* at 35.2 and 17.0% yields, respectively (calculated based on dry weight). Each of the extracts was shaken with n-hexane to remove much of the volatile oils and fatty components, and the resultant extract was then subjected to antioxidant assay.

### 2.3. Isolation of Curcuminoids 

The defatted rhizome extract (14 g) of *C. domestica* was fractionated by vacuum liquid chromatography (VLC) on silica gel type H (10–40 *μ*m, 7 × 30 cm) and eluted with a gradient system of hexane : CHCl_3_ (10 : 0–1 : 9, v/v) and CHCl_3_ : MeOH (10 : 0–0 : 10, v/v); repeated silica gel column (40–63 *μ*m, 3 × 60 cm) was eluted consecutively with toluene : CHCl_3_ : MeOH (75 : 15 : 5, v/v) and a gradient system of hexane : EtOAc (10 : 0–1 : 9, v/v) followed by recrystallization from EtOAc : hexane to yield curcumin (500 mg, 3.6%), demethoxycurcumin (200 mg, 1.4%), and bisdemethoxycurcumin (300 mg, 2.1%). The procedure for isolation of the compounds from *C. xanthorrhiza* was similar to that performed on *C. domestica. *Thirteen g of the defatted methanol extract of *C. xanthorrhiza* resulted in the isolation of curcumin (300 mg, 2.3%), demethoxycurcumin (250 mg, 1.9%), and bisdemethoxycurcumin (100 mg, 0.8%).

#### 2.3.1. Curcumin

Orange crystals, mp 184°C. HRESIMS: *m/z*: 391.0585 [M + Na]^+^ 759.1357 [2M + Na]^+^ which corresponded to C_21_H_20_O_6_. NMR ^1^H (acetone, 600 MHz): *δ* 7.60 (2H, *d*, *J* = 15.6 Hz, H-1, H-7), 7.32 (2H, *d*, *J* = 1.8 Hz, H-9, H-15), 7.16 (2H, *dd*, *J* = 1.8, 8.0, 1.8 Hz, H-13, H-19), 6.88 (2H, *d*, *J* = 7.8 Hz, H-12, H-18), 6.70 (2H, *d*, *J* = 16.2 Hz, H-2, H-6), 5.96 (1H, *s*, H-4), 3.91 (6H, *s*, OMe); NMR ^13^C (acetone, 600 MHz): *δ* 55.5 (C-OMe), 100.7 (C-4), 110.8 (C-9, C-15), 115.4 (C-12, C-18), 121.5 (C-2, C-6), 122.9 (C-13, C-19), 127.3 (C-8, C-14), 140.5 (C-1, C-7), 147.9 (C-10, C-16), 149.2 (C-11, C-17), 183.6 (C-3, C-5).

#### 2.3.2. Demethoxycurcumin

Orange crystals, mp 172°C. Its molecular formula was C_20_H_18_O_5_ as indicated by HRESIMS: *m/z*: 337.0826 [M-H]^−^, 675.1758 [2M-H]^−^. NMR ^1^H (acetone, 400 MHz): *δ* 7.62 (1H, *d*, *J* = 8.0 Hz, H-9), 7.59 (1H, *d*, *J* = 15.9 Hz, H-1), 7.58 (1H, *d*, *J* = 15.4 Hz, H-7), 7.56 (1H, *d*, *J* = 8.4 Hz, H-13), 7.34 (1H, *s*, H-15), 7.19 (1H, *d*, *J* = 8.0 Hz, H-19), 6.91 (3H, *d*, *J* = 8.4 Hz, H-10, H-12, H-18), 6.87 (1H, *d*, *J* = 16.4 Hz, H-2), 6.74 (1H, *d*, *J* = 16.1 Hz, H-6), 5.97 (1H, *s*, H-4), 3.91 (3H, *s*, OMe); NMR ^13^C (acetone, 400 MHz): *δ* 56.3 (C-OMe), 101.8 (C-4), 111.5 (C-15), 116.3 (C-18), 116.9 (C-10, C-12), 122.1 (C-2), 122.3 (C-6), 123.9 (C-19), 127.7 (C-8), 128.2 (C-14), 131.1 (C-9, C-13), 141.1 (C-7), 141.5 (C-1), 148.8 (C-17), 150.1 (C-16), 160.6 (C-11), 184.5 (C-5), 184.6 (C-3).

#### 2.3.3. Bisdemethoxycurcumin

Orange crystals, mp 222°C. HRESIMS for C_19_H_16_O_4_ *m/z*: 307.0757 [M-H]^−^. NMR ^1^H (acetone, 400 MHz): *δ* 7.61 (4H, *d*, *J* = 8.4 Hz, H-9, H-13, H-15, H-19), 7.55 (2H, *d*, *J* = 15.7 Hz, H-1, H-7), 6.86 (4H, *d*, *J* = 8.0 Hz, H-10, H-12, H-16, H-18), 6.73 (2H, *d*, *J* = 15.8 Hz, H-2, H-6); NMR ^13^C (acetone, 400 MHz): *δ* 100.7 (C-4), 116.0 (C-10, C-12, C-16, C-18), 121.4 (C-2, C-6), 127.4 (C-9, C-13, C-15, C-19), 130.2 (C-8, C-14), 140.6 (C-1, C-7), 161.3 (C-11, C-17), 183.5 (C-3, C-5). 

### 2.4. Preparation of Essential Oils

The fresh rhizomes of *C. domestica* and *C. xanthorrhiza* were washed, comminuted, and hydrodistilled in Clevenger-type apparatus for 8 h. The oily layers obtained were separated and dried over anhydrous magnesium sulphate. The percentage yields of oils based on dry weight for *C. domestica* and *C. xanthorrhiza* were 2.9 and 4.5%, respectively.

### 2.5. Analysis of the Essential Oils

Gas chromatography (GC) and gas chromatography-mass spectrometry (GC-MS) analyses were used for the identification of the essential oil components. The components were separated using a Shimadzu GC-2010 equipped with a flame ionizing detector (FID) and a DB-5 (30 m × 0.25 mm, 1 *μ*m film thickness) capillary column. One *μ*L of each sample, dissolved in ethyl acetate, was injected automatically in spilt mode (autoinjector Shimadzu AOC-20i), using pressure-controlled nitrogen as a carrier gas at a linear velocity of 50 cm^3^/min. The temperature of the injector and the detector was maintained at 250°C. The oven temperature was programmed from 75°C for 10 min, then at 3°C/min to 250°C and held for 5 min. The oils were also examined using stationary phase SE-30 (30 m × 0.25 mm, 0.25 *μ*m film thickness) under the following program conditions; initial temperature 60°C for 10 min, then 3°C/min to 230°C for 1 min. Peak areas and retention times were measured by computerized integration. The relative amounts of individual components were calculated based on the peak areas obtained without a flame ionization detector (FID) response factor correction. The linear retention indices of the components relative to n-alkanes were also determined. The oils were also analyzed using a Hewlett Packard GC-MSD 5890 series II; EI electron impact mode with electron energy 70 eV, scan time 1.5 s, and mass range 40–500 Da using a BPX5 (25 m × 0.25 mm × 0.25 *μ*m film thickness) capillary column. Similar conditions were used as described in GC programs. Components were identified by comparing their relative retention indices with those in the literature, their mass spectral data with the existing Wiley library, and cochromatography of some components with authentic components on the DB-5 capillary column [13]. 

### 2.6. Isolation of Pure Compounds from the Essential Oils 

The essential oils of *C. xanthorrhiza* and *C. domestica* were subjected to repeated column chromatography on silica gel (230–400 mesh) eluted with hexane-ethyl acetate (1 : 1, v/v), hexane-ethyl acetate (3 : 7, v/v), and 100% ethyl acetate. Xanthorrhizol in 18% yield and ar-turmerone in 30% yield were obtained from the essential oils of *C. xanthorrhiza* and *C. domestica*, respectively. The compounds were identified by spectroscopic techniques and by comparison with published data [14, 15].

### 2.7. Human LDL Isolation 

The use of human whole blood in this study was approved by the Ethics Committee of Universiti Kebangsaan Malaysia (UKM) (approval no. FF-120-2007). All subjects were healthy volunteers aged 24–70 yrs, normolipidemic, nonsmokers, having not taken any medications including vitamin supplements within the last 2 weeks, and fasting for the last 8 h. Venous blood was drawn from the volunteers, and 9 volumes of blood were added into 1 volume of 3.8% (w/v) sodium citrate solution as an anticoagulant. Plasma was obtained by centrifugation at 2000 g for 20 min. LDL was isolated by density gradient ultracentrifugation using a method developed by Graham et al. [16] with slight modification using OptiPrep as the density gradient medium. Briefly, 3.2 mL of plasma was mixed with 0.8 mL of OptiPrep (60% iodixanol) to give a final iodixanol concentration of 12% (v/v); 4 mL of this was layered under 4 mL of 6% iodixanol in saline in an 8.9 mL Opti Seal tube [17]. The tube was topped up with saline and ultracentrifuged at 402 000 g at 16°C for 3 h 10 min in a Ti. 70.1 rotor. The subfractions of lipoprotein were labelled as VLDL (very low-density lipoprotein), LDL, GAP (mixture of LDL and HDL), and HDL (high-density lipoprotein). The brightly coloured LDL band was located approximately one-third of the way down the tube and was isolated using a pasteur pipette. LDL was characterized by measuring the amount of protein by the Bradford protein assay using bovine serum albumin as standard [18]. LDL was diluted with phosphate-buffered saline (PBS)(pH 7.4) to a final concentration of 200 *μ*g protein/mL prior to oxidation analysis. The purity of LDL was evaluated by using a UV spectrophotometer as described by Galle and Wanner [19] and agarose gel electrophoresis as described by Noble [20]. The electrophoretic mobility of LDL was measured using agarose gels. Samples were electrophoresed at a constant of 45 mA/gel for 45 min, then oven dried at 85°C and stained with Sudan Black for 20 min. 

### 2.8. Oxidation of LDL

LDL (200 *μ*g protein/mL) was oxidised by exposing it to 10 *μ*M CuSO_4_ at 37°C for 5 h [17]. This incubation was also carried out in the presence of serial dilutions (5, 2.5, 1.25, 0.63, 0.31, and 0.16 *μ*g/*μ*L) of each sample in DMSO. The oxidation of LDL was terminated by rapid freezing. Samples intended for TBARS (thiobarbituric acid reactive substances) analysis were kept at −20°C for a maximum of 48 h. The methanol extracts and essential oils of *C. xanthorrhiza* and *C. domestica, *isolated curcuminoids (curcumin, demethoxycurcumin, and bisdemethoxycurcumin) and the essential oil standards (xanthorrhizol, ar-turmerone, ar-curcumene, zerumbone, camphor *β*-curcumene, zingiberene, and geranyl acetate), and probucol (as a positive control) were added to LDL directly before incubation.

### 2.9. TBARS Assay

The inhibition of copper-catalysed LDL oxidation was determined using TBARS assay [21]. Five *μ*L of the sample was added to a cuvette containing 945 *μ*L of LDL and 50 *μ*L of CuSO_4_ and incubated at 37°C for 5 h. A mixture containing LDL and CuSO_4_ was used as control, and blank experiment consisted of LDL and 0.5% DMSO. The total volume of the mixture was 1 mL. The final concentrations of the sample in the mixture were 25.0, 12.5, 6.25, 1.13, 1.56, and 0.78 *μ*g/mL. Further dilutions were carried out for active samples to obtain the final concentrations of 0.39, 0.20, and 0.10 *μ*g/mL. Probucol was used as the positive control in the assay. The final concentration of DMSO in the reaction mixtures was less than 0.5% to eliminate the effect of the solvent on the reaction as evidenced by control experiments. After the incubation, sodium dodecyl sulphate (SDS) and thiobarbituric acid (TBA) were added to the mixture followed by incubation at 95°C for 1 h to increase the peroxidation. The mixture was bathed with ice for 10 min to cool down and stop the peroxidation process. The precipitate formed was removed by centrifugation at 3000 rpm for 15 min. Malondialdehyde (MDA) in the supernatant was determined at 532 nm. TBARS are expressed in terms of MDA equivalents, and the results are expressed as nmoles of MDA/mg LDL protein. MDA standard was used to construct a standard curve [17]. 

The percentage inhibition of LDL oxidation was calculated as follows:

(1) $$\begin{array}{c} 1-\left(\frac{\text{oxidation}\;\;\text{of}\;\;\text{sample}}{\text{oxidation}\;\;\text{of}\;\;\text{control}}\right)\times 100. \end{array}$$

### 2.10. Statistical Analysis

All the data are presented as means ± standard error median (SEM) from triplicate experiments and were analysed using *Statistical Package for the Social Sciences* (SPSS) software version 17.0. A one-way analysis of variance (ANOVA) was used for multiple comparison. The concentration of the compounds required to inhibit 50% oxidation (IC_50_) for active extract was determined using Probit programme. *P* < 0.05 was considered to be statistically significant.

## 3. Results and Discussion 

### 3.1. Isolation and Identification of Curcuminoids from *C. domestica *and* C. xanthorrhiza*

It was found that *Curcuma domestica* has higher levels of total curcuminoids (7.1%) compared to *C. xanthorrhiza* (5.0%). The high percentage of curcuminoids isolated from both plants indicates that they are good sources for the isolation of curcuminoids. Curcumin was found to be the major compound in both species, where its concentration was higher in *C. domestica* (3.6%) than in *C. xanthorrhiza* (2.3%). Bisdemethoxycurcumin was also present at higher concentration in *C. domestica* (2.1%) than in *C. xanthorrhiza* (0.8%), but the latter contained higher amount of demethoxycurcumin (1.9%). The purity of the isolated compounds were confirmed by melting point determination. The structures of the compounds were elucidated by spectroscopic techniques including NMR and MS spectroscopy and confirmed by comparison with the literature values [22]. 

### 3.2. Chemical Composition of the Essential Oils

The chemical composition of the essential oils of *Curcuma xanthorrhiza* and *C. domestica* which showed strong inhibitory activity on LDL peroxidation was investigated in an effort to correlate the constituents of the oils and their antioxidant activity. The list of constituents identified in the oils is shown in Table 1 in order of elution on a DB-5 type column. The chemical components of the essential oils of* Curcuma xanthorrhiza* and *C. domestica* have been previously reported by us [4]. A comparison between the oils of the present study, with those reported by us previously showed that more compounds have been identified in the present study and there were some compositional differences and considerable variation in the levels of some individual constituents. The rhizome oil of *C. xanthorrhiza *was characterised by the presence of a high concentration of bisabolene-type sesquiterpenes and their oxygenated derivatives which accounted for more than 92% of the oil. The most abundant component was the sesquiterpene phenol, xanthorrhizol (32%). The other major compounds present in the oil were *β*-curcumene (17.1%), zingiberene (13.2%), *β*-bisabolol (3.5%), and ar-curcumene (2.6%). The major components of the rhizome oils of *C. domestica* were ar-turmerone (45.8%), curcumenol (18.2%), and geranyl acetate (2.5%). The chemical composition of the oil was qualitatively similar to those reported by other workers, although there were some variations in the composition and levels of individual constituents of the oils, suggesting the existence of chemical varieties [2, 3, 9]. However, the variations may also be due to environmental factors such as the growth conditions and postharvest handling and processing.

### 3.3. LDL Antioxidant Activity

The methanol extracts and essential oils of *Curcuma domestica* and *C. xanthorrhiza* were investigated for their ability to inhibit copper-mediated oxidation on isolated human LDL. The methanol extracts of *C. xanthorrhiza *and *C. domestica* showed strong inhibition of LDL oxidation at 6.25 *μ*g/mL, exhibiting greater than 84% inhibition (Table 2). The essential oils of the plants showed weaker activity than their methanol extracts, exhibiting >78% inhibition at 25.0 *μ*g/mL (Table 3). The results demonstrated that the methanol extracts and the oils inhibited copper-mediated oxidation of LDL in a dose-dependent manner; that is, as the concentration of the samples increased, the percentage inhibition of LDL peroxidation increased. The IC_50_ values of the methanol extracts and the oils with LDL antioxidant activity are shown in Tables 2 and 3.

 The antioxidant activity of the isolated curcuminoids (curcumin, demethoxycurcumin, and bisdemethoxycurcumin) and the 8 standard compounds (xanthorrhizol, ar-turmerone, ar-curcumene, *β*-curcumene, zerumbone, zingiberene, camphor, and geranyl acetate) that are the major components of the oils were also investigated. The three curcuminoids showed strong inhibition on LDL peroxidation, with curcumin and demethoxycurcumin showing comparable antioxidant activity and more potent than bisdemethoxycurcumin (Table 2). The three curcuminoids showed strong inhibition on LDL peroxidation with IC_50_ values of 0.15, 0.16, and 0.36 *μ*mol/L, respectively, lower than that of probucol (0.57 *μ*mol/L), a potent inhibitor of copper-catalysed LDL peroxidation [23]. The high antioxidant activity of the three curcuminoids was consistent with previous results obtained using three different bioassay models, that is, the linoleic acid auto-oxidation model, rabbit erythrocyte membrane ghost system, and liver microsome system [7]. The present study was in accordance with previous studies which indicated that the absence of one methoxy group (demethoxycurcumin) on the phenyl ring did not have effect, but the absence of both methoxy groups (bisdemethoxycurcumin) resulted in decreased antioxidant activity in curcuminoids. The phenolic hydroxyl and the methoxyl groups on the phenyl ring and the 1,3-diketone system are important structural features for antioxidant activity [6].

 Xanthorrhizol was found to be the most active compound in the oil of *C. xanthorrhiza*, with IC_50_ value of 1.93 *μ*mol/L (Table 3). Ar-turmerone and zerumbone were the compounds in the oil of *C. domestica* that exhibited strong inhibition of LDL peroxidation, with IC_50_ values of 10.18 and 24.90 *μ*mol/L, respectively. The other compounds, ar-curcumene, camphor, and geranyl acetate, showed relatively weak activity. The inhibition of LDL peroxidation by xanthorrhizol, ar-turmerone, and zerumbone was dose dependant. Structure-activity analysis indicated that nonoxygenated bisabolene-type sesquiterpenes exhibited weak antioxidant activity. The strong antioxidant effect of xanthorrhizol is most likely due to the presence of a phenolic hydroxyl group on the bisabolene skeleton. The antioxidant effect appears to be due to the ability of the compounds to chelate Cu^2+^ ion and thus may inhibit the initiation of LDL oxidation and free radical formation at the lipoprotein [24].

### 3.4. Correlation between Antioxidant Activity and the Chemical Contents

Based on the results of the LDL antioxidant assays on the methanol extracts, essential oils, isolated compounds, and standard samples, relationships between the antioxidant activity and the chemical contents can be deduced. The results suggest that the strong antioxidant activity of the methanol extracts of the plants is due to the presence of high amounts of curcuminoids. The higher activity of the methanol extract of *C. domestica* (IC_50_, 0.31 *μ*g/mL) than *C. xanthorrhiza *(IC_50_, 0.78 *μ*g/mL) could possibly be due to the higher level of curcuminoids in the former (Table 2). The strong antioxidant activity of the rhizome oil of *C. xanthorrhiza *may be related to the high level of xanthorrhizol (32%), although other constituents may also contribute to the antioxidant activity of the oil (Table 3). Previous studies have indicated that xanthorrhizol strongly inhibited platelet aggregation induced by arachidonic acid, collagen, and ADP [25] and had antimicrobial activity against *Candida* species, filamentous fungi and food-borne pathogens, *Staphylococcus aureus* and methicillin-resistant *Staphylococcus aureus, *(MRSA) [26, 27]. It also nonselectively inhibited DNA, RNA, and protein synthesis and exhibited antiproliferative activity on different cancer cell lines [28]. The presence of high levels of ar-turmerone (45.8%) and zerumbone (1.4%) in the rhizome oil of *C. domestica* could explain its strong inhibition on LDL peroxidation. The strong antioxidant activity of the oil may be possibly due to the synergy between these compounds with the other constituents of the oil. Previous studies have indicated that ar-turmerone isolated from *C. longa* inhibited collagen- and arachidonic-induced platelet aggregation [29], exhibited immunomodulatory activity [30], and induced the apoptotic activity in various cell lines [31]. Antitumor activity of zerumbone isolated from *Zingiber zerumbet *against various cancer cell lines has been widely investigated [32, 33]. Zerumbone has also showed anti-inflammatory and antinociceptive activities [34]. 

## 4. Conclusion 

The methanol extracts and essential oils of *C. xanthorrhiza *and* C. domestica *showed high LDL antioxidant activity. The high antioxidant activity of the methanol extracts of the plants could be due to the high amounts of curcuminoids present. The high levels of xanthorrhizol and ar-turmerone in the rhizome oils of *C. xanthorrhiza* and *C. domestica*, respectively, could explain their strong inhibition on LDL peroxidation. 

## Figures and Tables

**Table 1:** Chemical constituents of the rhizome oils of *Curcuma domestica* and *Curcuma xanthorrhiza*.

Compound	Percentage		RI	Method of identification
	*C. * *domestica *	*C. * *xanthorrhiza *		
*α*-Thujene	—	tr	931	a, b
*α*-Pinene	—	0.3	940	a, b, c
Camphene	—	0.7	956	a, b, c
*β*-Pinene	—	0.1	976	a, b, c
Cis-Pinane	—	0.1	986	a
Myrcene	—	0.1	991	a, b, c
*α*-Phellandrene	1.1	tr	1009	a, b, c
*α*-Terpinene	0.2	0.1	1016	a, b, c
p-Cymene	0.2	—	1029	a, b, c
1,8-Cineole	0.3	0.1	1034	a, b, c
(*Z*)-*β*-Ocimene	—	0.1	1037	a, b, c
*γ*-Terpinene	—	tr	1063	a, b, c
Terpinolene	0.3	—	1090	a, b, c
6,7-Epoxymyrcene	—	tr	1093	a
Camphor	—	5.4	1156	a, b, c
Cis-dehydro-*β*-terpineol	—	0.3	1160	a, b
*α*-Terpineol	—	0.3	1167	a, b, c
Terpinen-4-ol	—	0.2	1177	a, b, c
Ethyl-4E-octenoate	—	0.1	1187	a
Dihydro citronellol acetate	—	0.1	1321	a
*α*-Cubebene	—	0.1	1351	a, b
(*Z*)-*β*-Damascenone	—	0.1	1364	a
n-Undecanol	—	tr	1370	a, b
Geranyl acetate	2.5	tr	1382	a, b, c
*β*-Cubebene	—	tr	1388	a, b
*β*-Elemene	1.5	—	1389	a, b
Methyl perillate	—	0.1	1394	a
(*Z*)-Isoeugenol	—	0.2	1407	a, b, c
*α*-Cis-bergamotene	0.9	0.6	1414	a, b
Methyl undecanoate	—	0.1	1428	a
*β*-Humulene	—	0.1	1439	a, b, c
(*Z*)-*β*-Farnesene	—	0.2	1443	a, b
(*E*)-caryophyllene	0.3	—	1444	a, b, c
*γ*-elemene	—	0.4	1448	a, b
(*E*)-*β*-farnesene	0.2.0	1.2	1457	a, b
*α*-Humulene	0.1	—	1460	a
(*E*)-Ethyl cinnamate	—	tr	1467	a, b, c
Ar-Curcumene	1.2	13.2	1493	a, b, c
*γ*-Curcumene	0.4	2.6	1481	a, b
*β*-Bisabolene	0.2	0.6	1505	a, b
Zingiberene	1.7	—	1506	a, b, c
(*Z*)-*γ*-Bisabolene	—	2.6	1516	a, b
*β*-Curcumene	0.8	17.1	1523	a, b, c
*β*-Sesquiphellandrene	1.9	0.4	1538	a, b
1,10-Decanediol	—	0.4	1549	a, b
(*Z*)-Isoeugenol acetate	0.3	1.2	1567	a, b
Ar-Turmerol	0.3	—	1571	a, b, c
Caryophyllene oxide	0.3	0.5	1581	a, b, c
Thujopsan-2-*α*-ol	—	0.3	1587	a
Ar-Dihydro-turmerone	1.0	—	1592	a, b
Sesquithuriferol	—	0.2	1605	a
Geranyl isovalerate	0.3	—	1607	a
1,10-Di-epi-cubenol	—	0.4	1619	a
(*Z*)-Dihydromyrcene-1,6-diol	3.6	—	1621	a, b
10-Epi-*γ*-eudesmol	0.4	—	1624	a
Citronellyl pentanoate	—	5.7	1626	a, b
1-Epi-cubenol	0.8	—	1629	a
*γ*-Eudesmol	0.2	—	1632	a, b
Cis-cadin-4-en-7-ol	—	0.8	1637	a
Hinesol	0.4	—	1642	a, b
Cubenol	0.3	0.5	1647	a, b
*β*-Eudesmol	1.3	—	1651	a, b
*α*-Eudesmol	—	0.8	1654	a, b
(*E*)-Dihydromyrcene-1,6-diol	1.3	—	1655	a, b
(*E*)-Amyl cinnamic alcohol	—	0.7	1661	a
14-Hydroxy-9-epi-(*E*)-caryophyllene	0.4	—	1670	a
(*E*)-citronellyl tiglate	—	0.9	1668	a
*β*-Bisabolol	—	3.5	1687	a, b
Ar-Turmerone	45.8	—	1689	a, b, c
Ar-Curcumen-15-al	—	0.8	1712	a
14-Hydroxy-*α*-humulene	0.2	—	1714	a
1-Phenyl-hepta-1,3,5-triyne	—	0.3	1721	a
Curcumenol	18.2	—	1724	a, b
4-Hydroxy-3-methoxy-cinnamaldehyde	—	0.9	1728	a
Zerumbone	1.4	—	1730	a, b, c
Chamazulene	—	0.3	1732	a
(*E*, *Z*)-Farnesol	—	0.1	1745	a, b
*α*-Bisabolol oxide A	—	0.2	1749	a
*α*-Amyl-cinnamyl acetate	0.2	—	1758	a
*β*-Bisabolen-12-ol	0.3	—	1762	a
*γ*-Curcumen-15-al	1.1	—	1767	a, b
Xanthorrhizol	—	31.9	1768	a, b, c
*γ*-Eudesmol acetate	1.6	—	1782	a, b
Butyl dodecanoate	—	0.2	1786	a
8-Cedren-14-ol acetate	0.8	—	1789	a, b
*α*-Eudesmol acetate	2.3	—	1795	a, b

Total	96.3	97.7		

Percentages were calculated on the basis of results obtained on column DB 5; RI: retention index; a: retention index; b: mass fragmentation; c: co-chromatography with authentic samples.

**Table 2:** Percentage inhibition and IC_50_ value (*μ*g/mL) of the methanol extracts and curcuminoids of *Curcuma domestica* and *C. xanthorrhiza* on human LDL peroxidation.

Sample	Concentration (*μ*g/mL)	Inhibition (%)	IC_50_ (*μ*g/mL)
	6.25	84.14 ± 0.13	
	3.13	83.77 ± 0.13	
*Curcuma domestica *	1.56	73.18 ± 0.78	0.31 ± 0.01
	0.78	68.73 ± 0.79	
	0.39	50.45 ± 0.20	

	12.5	91.94 ± 0.92*	
	6.25	88.04 ± 0.52	
*Curcuma xanthorrhiza *	3.13	77.82 ± 0.92	0.78 ± 0.03
	1.56	62.23 ± 0.13	
	0.78	50.89 ± 1.05	

	1.56	98.81 ± 0.10*	
	0.78	93.57 ± 0.56	
Demethoxycurcumin	0.39	84.23 ± 1.38	0.11 ± 0.01 (0.16 *μ*M)
	0.20	64.42 ± 3.10	
	0.10	47.83 ± 3.25	

	1.56	93.20 ± 0.59*	
	0.78	88.71 ± 0.98	
Bisdemethoxycurcumin	0.39	80.87 ± 1.67	0.05 ± 0.01 (0.33 *μ*M)
	0.20	71.90 ± 2.45	
	0.10	56.01 ± 0.71	

	1.56	90.58 ± 0.82*	
	0.78	89.09 ± 0.95	
Curcumin	0.39	78.25 ± 1.90	0.06 ± 0.01 (0.15 *μ*M)
	0.20	58.15 ± 2.89	
	0.10	50.39 ± 3.43	

	5.00	76.3 ± 0.6	
	2.50	74.2 ± 0.2	
Probucol	1.25	70.3 ± 0.1	0.30 ± 0.1 (0.57 *μ*M)
	0.63	62.1 ± 1.6	
	0.31	48.9 ± 0.1	

Probucol was used as a positive control. Values are presented as mean ± SEM (*n* = 3). *P < 0.05 as compared with the control. IC_50_ values in *μ*M are in parentheses.

**Table 3:** Percentage inhibition and IC_50_ value (*μ*g/mL) of the essential oils of *Curcuma* *domestica* and *C. xanthorrhiza* and the essential oil standards on human LDL peroxidation.

Sample	Concentration (*μ*g/mL)	Inhibition (%)	IC_50_ (*μ*g/mL)
	25	78.8 ± 1.2*	
	12.5	56.4 ± 1.0	
*Curcuma domestica *	6.25	44.8 ± 0.4	7.8 ± 0.2
	3.13	27.5 ± 1.5	
	1.56	20.0 ± 0.8	

	25	86.9 ± 2.3*	
	12.5	83.2 ± 1.2	
*Curcuma xanthorrhiza *	6.25	64.0 ± 1.3	2.2 ± 0.1
	3.13	57.8 ± 4.6	
	1.56	49.4 ± 0.2	

	12.5	98.0 ± 0.7*	
	6.25	87.5 ± 2.0	
Xanthorrhizol	3.13	84.5 ± 2.5	0.4 ± 0.1 (1.9 *μ*M)
	1.56	77.5 ± 0.8	
	0.78	64.6 ± 1.7	

	12.5	85.6 ± 0.7*	
	6.25	72.8 ± 1.0	
Ar-Turmerone	3.13	62.2 ± 1.7	2.2 ± 0.1 (10.2 *μ*M)
	1.56	41.7 ± 1.2	
	0.78	24.6 ± 0.8	

	25	69.9 ± 0.3*	
	12.5	58.1 ± 0.3	
Zerumbone	6.25	55.9 ± 0.7	5.4 ± 0.4 (24.9 *μ*M)
	3.13	49.0 ± 0.3	
	1.56	27.6 ± 2.9	

Geranyl acetate	25	26 ± 2.6	
Zingiberene	25	16 ± 1.9	
Ar-Curcumene	25	4.6 ± 1.2	—
*β*-Curcumene	25	7.8 ± 1.5	
Camphor	25	11.2 ± 0.4	—

Values are presented as mean ± SEM (*n* = 3). *P < 0.05 as compared with the control. IC_50_ values in *μ*M are in parentheses.

## References

[1] Burkill IH (1966). *A Dictionary of Economic Products of the Malay Peninsula*.

[2] Zwaving JH, Bos R (1992). Analysis of the essential oils of five *curcuma* species. *Flavour and Fragrance Journal*.

[3] Sharma RK, Misra BP, Sarma TC, Bordoloi AK, Pathak MG, Leclercq PA (1997). Essential Oils of *curcuma longa* L. from Bhutan. *Journal of Essential Oil Research*.

[4] Jantan IB, Ahmad AS, Ali NAM, Ahmad AR, Ibrahim H (1999). Chemical composition of the rhizome oils of four *curcuma* species from Malaysia. *Journal of Essential Oil Research*.

[5] Nishiyama T, Mae T, Kishida H (2005). Curcuminoids and sesquiterpenoids in turmeric (*curcuma longa* L.) suppress an increase in blood glucose level in type 2 diabetic KK-A*γ* mice. *Journal of Agricultural and Food Chemistry*.

[6] Jayaprakasha GK, Jagan Mohan Rao L, Sakariah KK (2005). Chemistry and biological activities of *C. longa*. *Trends in Food Science and Technology*.

[7] Itokawa H, Shi Q, Akiyama T, Morris-Natschke SL, Lee KH (2008). Recent advances in the investigation of curcuminoids. *Chinese Medicine*.

[8] Zhao J, Zhang JS, Yang B, Lv GP, Li SP (2010). Free radical scavenging activity and characterization of sesquiterpenoids in four species of *curcuma* using a TLC bioautography assay and GC-MS analysis. *Molecules*.

[9] Singh G, Kapoor IPS, Singh P, de Heluani CS, de Lampasona MP, Catalan CAN (2010). Comparative study of chemical composition and antioxidant activity of fresh and dry rhizomes of turmeric (*curcuma longa* Linn.). *Food and Chemical Toxicology*.

[10] Chen WF, Deng SL, Zhou B (2006). Curcumin and its analogues as potent inhibitors of low density lipoprotein oxidation: H-atom abstraction from the phenolic groups and possible involvement of the 4-hydroxy-3-methoxyphenyl groups. *Free Radical Biology and Medicine*.

[11] Wei Q-Y, Chen W-F, Zhou B, Yang L, Liu ZL (2006). Inhibition of lipid peroxidation and protein oxidation in rat liver mitochondria by curcumin and its analogues. *Biochimica et Biophysica Acta*.

[12] Wong SP, Leong LP, William Koh JH (2006). Antioxidant activities of aqueous extracts of selected plants. *Food Chemistry*.

[13] Adams RP (1989). *Identification of Essential Oil Components by Gas Chromatography/Mass Spectroscopy*.

[14] Hong CH, Kim Y, Lee SK (2001). Sesquiterpenoids from the Rhizome of *curcuma zedoaria*. *Archives of Pharmacal Research*.

[15] Sirat HM, Hong NM, Jauri MH (2007). Chemistry of xanthorrhizol: synthesis of several bisabolane sesquiterpenoids from xanthorrhizol. *Tetrahedron Letters*.

[16] Graham JM, Higgins JA, Gillott T (1996). A novel method for the rapid separation of plasma lipoproteins using self-generating gradients of iodixanol. *Atherosclerosis*.

[17] Dillon SA, Burmi RS, Lowe GM, Billington D, Rahman K (2003). Antioxidant properties of aged garlic extract: an in vitro study incorporating human low density lipoprotein. *Life Sciences*.

[18] Bradford MM (1976). A rapid and sensitive method for the quantitation of microgram quantities of protein utilizing the principle of protein dye binding. *Analytical Biochemistry*.

[19] Galle J, Wanner C (1998). Oxidized LDL and Lp(a) preparation, modification, and analysis. *Methods in Molecular Biology*.

[20] Noble RP (1968). Electrophoretic separation of plasma lipoproteins in agarose gel. *Journal of Lipid Research*.

[21] Buege JA, Aust SD (1978). Microsomal lipid peroxidation. *Methods in Enzymology*.

[22] Paramasivam M, Poi R, Banerjee H, Bandyopadhyay A (2009). High-performance thin layer chromatographic method for quantitative determination of curcuminoids in *curcuma longa* germplasm. *Food Chemistry*.

[23] Parthasarathy S, Young SG, Witztum JL (1986). Probucol inhibits oxidative modification of low density lipoprotein. *Journal of Clinical Investigation*.

[24] Yu L, Zhou K, Parry JW (2005). Inhibitory effects of wheat bran extracts on human LDL oxidation and free radicals. *LWT-Food Science and Technology*.

[25] Jantan I, Raweh SM, Sirat HM (2008). Inhibitory effect of compounds from Zingiberaceae species on human platelet aggregation. *Phytomedicine*.

[26] Rukayadi Y, Hwang JK (2007). *In Vitro* antimycotic activity of xanthorrhizol isolated from *curcuma xanthorrhiza* Roxb. against opportunistic filamentous fungi. *Phytotherapy Research*.

[27] Mustaffa F, Indurkar J, Ismail S, Shah M, Mansor SM (2011). An antimicrobial compound isolated from *Cinnamomum iners* leaves with activity against methicillin-resistant *Staphylococcus aureus*. *Molecules*.

[28] Cheah YH, Nordin FJ, Tee TT (2008). Antiproliferative property and apoptotic effect of xanthorrhizol on MDA-MB-231 breast cancer cells. *Anticancer Research*.

[29] Lee HS (2006). Antiplatelet property of *curcuma longa* L. rhizome-derived ar-turmerone. *Bioresource Technology*.

[30] Yue GGL, Chan BCL, Hon PM (2010). Evaluation of in vitro anti-proliferative and immunomodulatory activities of compounds isolated from *curcuma longa*. *Food and Chemical Toxicology*.

[31] Ji M, Choi J, Lee J, Lee Y (2004). Induction of apoptosis by ar-turmerone on various cell lines. *International journal of molecular medicine*.

[32] Adbul ABH, Al-Zubairi AS, Tailan ND (2008). Anticancer activity of natural compound (Zerumbone) extracted from *Zingiber zerumbet* in human HeLa cervical cancer cells. *International Journal of Pharmacology*.

[33] Kirana C, McIntosh GH, Record IR, Jones GP (2003). Antitumor activity of extract of *Zingiber aromaticum* and its bioactive sesquiterpenoid zerumbone. *Nutrition and Cancer*.

[34] Sulaiman MR, Perimal EK, Akhtar MN (2010). Anti-inflammatory effect of zerumbone on acute and chronic inflammation models in mice. *Fitoterapia*.

